# Mesenchymal Stem Cell-Mediated Targeted Drug Delivery Systems for Hepatocellular Carcinoma: Current Advances and Future Directions

**DOI:** 10.3390/bioengineering12111206

**Published:** 2025-11-04

**Authors:** Yang Gao, Jian-Ping Wang, De-Fei Hong, Chang Yang, Hua Naranmandura

**Affiliations:** 1 Department of Toxicology, School of Medicine and Public Health, Zhejiang University, Hangzhou 310058, China; 2 Department of General Surgery of The Affiliated Sir Run Run Shaw Hospital, School of Medicine, Zhejiang University, Hangzhou 310016, China; 3 Department of Hematology of First Affiliated Hospital, School of Medicine, Zhejiang University, Hangzhou 310058, China

**Keywords:** drug delivery, hepatocellular carcinoma, mesenchymal stem cells

## Abstract

Hepatocellular carcinoma (HCC) ranks as the second most lethal malignancy worldwide, presenting formidable therapeutic challenges including tumor heterogeneity, complex microenvironment, and inefficient drug delivery. Conventional therapies such as surgery, chemotherapy, and immunotherapy are limited by systemic toxicity, drug resistance, and poor targeting specificity. Mesenchymal stem cells (MSCs) have emerged as promising drug delivery vehicles, leveraging their innate tumor-homing capacity, immunomodulatory properties, and exosome-mediated cargo transport. Preclinical studies demonstrate that MSC-based systems triple drug accumulation in tumors and synergize with immunotherapy, extending survival in HCC models. This review systematically examines recent advances in MSC-based delivery systems for HCC, focusing on engineering strategies to enhance targeting precision and controlled drug release, including genetic modification, exosome engineering, and stimuli-response systems. Despite progress, challenges such as MSC heterogeneity and scalable production persist. Emerging solutions like single-cell RNA sequencing for subpopulation selection and 3D bioprinting for standardized culture are highlighted. This work provides a roadmap for developing MSC-based precision therapies, bridging translational gaps in HCC treatment.

## 1. Introduction

Hepatocellular carcinoma (HCC), representing 75–85% of primary liver cancers, ranks as the sixth most common malignant tumor globally [1,2]. According to the Global Burden of Disease Study, HCC patients face a five-year survival rate of only 12%, with mortality rates being the second highest among all cancers, surpassed only by pancreatic cancer [3,4]. Current clinical management of HCC relies on multidisciplinary strategies: curative therapies such as hepatic resection and liver transplantation are applicable to early-stage patients but suffer from a 70% five-year recurrence rate; locoregional therapies including transarterial chemoembolization (TACE), radiofrequency ablation, and radiotherapy show limited efficacy in advanced stages while risking damage to healthy liver tissue; systemic therapies involving targeted drugs like sorafenib and lenvatinib or immune checkpoint inhibitors such as PD-1/PD-L1 inhibitors are hindered by drug resistance, systemic toxicity, and low response rates [5,6]. These limitations stem primarily from tumor heterogeneity, the complex hepatic microenvironment, and off-target effects of conventional drug delivery systems. Therefore, innovative therapeutic strategies that integrate molecular mechanism elucidation, advanced drug delivery systems, and combinatorial treatment approaches are urgently needed to improve HCC treatment.

Driven by these challenges, the development of tumor-targeted delivery systems has emerged as a critical solution [7]. By precisely regulating tumor tropism, spatiotemporal drug release, and microenvironment-responsive behaviors, next-generation delivery platforms demonstrate multifunctional advantages [8]. For example, polyethylene glycolated liposomal doxorubicin enhances drug accumulation in HCC tissues through passive targeting, while reducing off-target cardiotoxicity and myelosuppression risks compared to conventional doxorubicin [9]. More sophisticated platforms like hypoxia/pH-responsive nanocarriers enable microenvironment-triggered sorafenib release, simultaneously inhibiting autophagy and reprogramming immunosuppressive niches to extend progression-free survival in preclinical models [10].

Mesenchymal stem cells (MSCs) stand out among innovative delivery platforms due to their unique therapeutic potential. First isolated from bone marrow in 1976, MSCs have since been identified in various human tissues as fibroblast-like, mesoderm-derived progenitor cells with multipotent differentiation capacity, and are defined by the International Society for Cellular Therapy as an adherent cell population expressing CD73, CD90, and CD105 while lacking CD34 and CD45, and can differentiate in vitro into osteoblasts, adipocytes, and chondrocytes [11,12].

MSCs demonstrate core advantages, including innate tumor-homing capabilities and immunomodulatory functions [13]. Unlike macrophage-based carriers, MSCs more effectively resist the immunosuppressive tumor microenvironment, maintaining stable homing and penetration efficiency into tumors. MSCs migrate to tumor sites via the CXCR4/SDF-1 signaling axis and remodel the immunosuppressive microenvironment by secreting TNF-α and IL-1β, thereby enhancing PD-1 antibody delivery efficiency [14]. Engineered MSCs can deliver MSC/IL-12 recombinant adenovirus and stimulate IFN-γ production, synergistically inhibiting tumor growth through multiple mechanisms [15,16]. Additionally, multivalent antibody engineering of MSCs significantly improves their homing efficiency to damaged tissues and therapeutic efficacy [17]. Furthermore, exosomes derived from miR-122-modified adipose-derived mesenchymal stem cells (ADSCs) suppress Cyclin G1 and ADAM10 expression in HCC cells, which reverses multidrug resistance and enhances chemosensitivity. In this review, we summarize recent advances in MSC-based targeted delivery systems and their therapeutic potential in overcoming the current limitations in hepatocellular carcinoma treatment.

**Figure 1:**
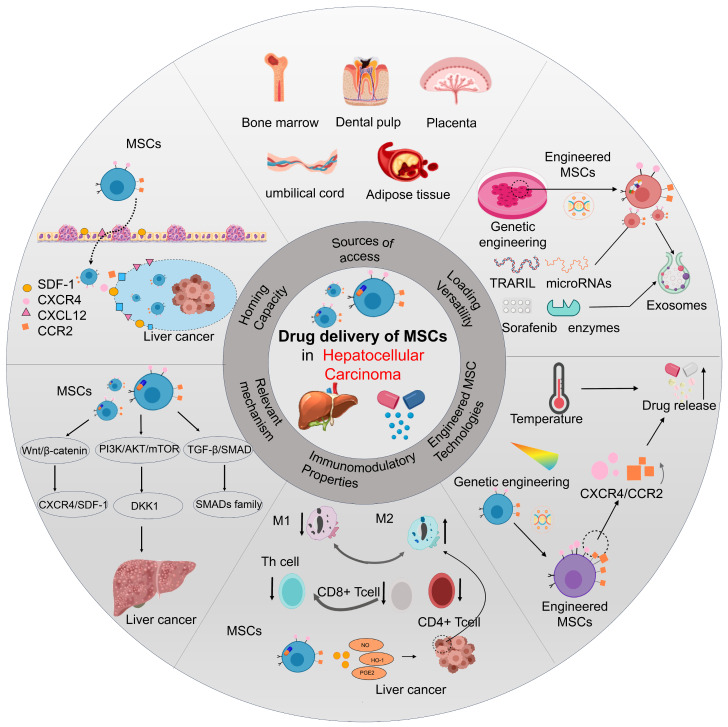
Schematic Diagram of the Characteristics of Mesenchymal Stem Cell. This schematic provides a structured overview of mesenchymal stem cell (MSC) features relevant to hepatocellular carcinoma (HCC) therapy. Arranged concentrically, the diagram details three core aspects: the outer ring showcases MSC sources (bone marrow, dental pulp, etc.) and their engineering versatility; the middle ring delineates the critical homing mechanisms and immunomodulatory functions mediating interactions within the tumor microenvironment; and the central circle emphasizes cutting-edge control systems like the LightOn technology.

The comprehensive strategy of utilizing mesenchymal stem cells as delivery vehicles for HCC therapy—encompassing cell sources, engineering approaches, homing mechanisms, and immunomodulatory effects—is conceptually summarized in Figure 1 (the overview diagram). This schematic provides a foundational framework for the detailed discussions of individual components in the following sections.

## 2. Current Landscape of Hepatocellular Carcinoma Therapy

The contemporary management of hepatocellular carcinoma (HCC) reflects an evolving therapeutic landscape that strategically integrates conventional modalities with innovative interventions. Surgical resection remains the cornerstone of curative-intent treatment, demonstrating optimal outcomes in early-stage HCC patients who meet Milan criteria, while also maintaining clinical relevance in select intermediate-stage cases. Notably, even patients with vascular invasion derive superior overall survival benefits from resection compared to those from systemic therapy alternatives [18,19]. Liver transplantation has emerged as a transformative radical treatment, particularly for early-stage HCC patients who are not surgical candidates. Its clinical utility continues to expand through two key developments: (1) refinement of downstaging protocols and (2) progressive liberalization of transplant eligibility criteria. Importantly, transplantation confers significant improvements in both event-free and overall survival, even for patients initially exceeding Milan criteria who achieve successful downstaging [20,21]. Beyond classical HCC indications, transplantation demonstrates curative potential in carefully selected cases, including perihilar cholangiocarcinoma patients who respond favorably to neoadjuvant therapy [22].

Conventional chemotherapeutic agents continue to play crucial roles in HCC management, with the primary therapeutic arsenal comprising platinum-based compounds such as oxaliplatin and cisplatin, fluorouracil derivatives, topoisomerase inhibitors including irinotecan and topotecan, and anthracycline-class drugs exemplified by doxorubicin [23,24]. These cytotoxic agents mediate their antitumor effects through diverse molecular mechanisms, among which the FOLFOX regimen—a combination of oxaliplatin, fluorouracil, and leucovorin—has demonstrated particular promise in enhancing surgical conversion rates for carefully selected patients with either resectable or advanced HCC [25]. Notably, when delivered via hepatic arterial infusion chemotherapy for cases complicated by portal vein tumor thrombosis, the FOLFOX protocol exhibits significantly superior therapeutic outcomes compared to sorafenib monotherapy [26]. While doxorubicin historically occupied a prominent position in early HCC treatment algorithms due to its demonstrable antitumor efficacy, its contemporary clinical application has experienced a substantial decline owing to dose-limiting cardiotoxic effects that compromise its safety profile [27].

Targeted therapies, such as anti-angiogenic agents, have been the mainstay of treatment for HCC through specifically inhibiting multiple mechanisms [28]. As the first approved targeted agent, sorafenib prolongs median overall survival to 10.7 months in advanced HCC by concurrently blocking VEGFR, PDGFR, and RAF/MEK/ERK pathways while uniquely generating reactive oxygen species through electron transport chain complex inhibition [29]. Lenvatinib, as an alternative first-line targeted therapy for HCC, has shown clinically superior outcomes compared to sorafenib, with a doubling of median progression-free survival (7.4 vs. 3.7 months), nearly triple the objective response rate (24% vs. 9%), and comparable overall survival (13.6 vs. 12.3 months) while achieving significantly higher disease control rates (66.7% vs. 22.2%) [30]. Lenvatinib’s multi-targeted inhibition of VEGFR, FGFR, and other kinases confers dual anti-angiogenic and tumor-immune microenvironment modulatory effects [31]. Despite the longstanding first-line status in advanced HCC management, their clinical utility remains constrained by pronounced resistance patterns encompassing both primary and acquired forms, modest survival benefits with low response rates, and differential efficacy favoring hepatitis B virus-related HCC over hepatitis C virus-associated cases [32,33]. Compounding these limitations, both sorafenib and lenvatinib face substantial delivery challenges stemming from tumor vascular abnormalities that impair drug permeability, cirrhotic liver dysfunction reducing bioavailability, and metabolic disturbances, collectively necessitating therapeutic regimen optimization and novel targeted strategy development [34].

Targeted therapy for hepatocellular carcinoma has evolved remarkably from the initial monotherapy era marked by sorafenib’s 2007 approval to the current paradigm of synergistic combination strategies. This evolution is exemplified by the breakthrough PD-1/PD-L1 inhibitor atezolizumab plus anti-VEGF bevacizumab regimen, which establishes a new first-line standard through dual mechanisms of immune checkpoint blockade and vascular normalization, achieving unprecedented survival benefits [35,36]. The IMbrave150 clinical trial demonstrated that the combination regimen significantly improved outcomes compared with sorafenib monotherapy, with a near tripling of the objective response rate (27.3% vs. 11.9%), a 58% prolongation of median progression-free survival (6.8 months vs. 4.3 months), and a higher one-year overall survival rate (67.2% vs. 54.6%) [37]. Other combination strategies, such as dual immune checkpoint blockade with durvalumab plus tremelimumab, also showed promising results, including an objective response rate of 31% and a median overall survival of 18.7 months [38]. Current combination therapies consistently achieve superior disease control rates, reaching 55–73%, compared to monotherapy [39]. Despite atezolizumab-bevacizumab becoming the first-line standard regimen for advanced hepatocellular carcinoma, its clinical application still faces notable limitations, including significantly increased incidence of treatment-related adverse events, such as grade 3–4 hypertension and proteinuria, as well as severe adverse events like hemorrhage and gastrointestinal perforation [40]. More critically, treatment-related fatalities, including life-threatening complications such as cholangitis and esophageal variceal bleeding, have been reported in the combination therapy group [41]. To address the critical challenges of drug resistance, insufficient targeting, adverse effects, and tumor heterogeneity in current hepatocellular carcinoma-targeted therapies, including immune-based combinations and epigenetic/metabolic-targeting drugs, there is an urgent need to develop novel drug delivery systems capable of achieving high tumor-specific accumulation and controlled release while minimizing off-target toxicity, thereby providing transformative solutions to overcome hepatocellular carcinoma treatment barriers [35,36,42,43].

## 3. Biological Basis of MSC-Based Delivery Systems

### 3.1. Source

Mesenchymal stem cells (MSCs) demonstrate remarkable tissue-specific diversity, with major sources including bone marrow (BM-MSCs), adipose tissue (AT-MSCs), umbilical cord Wharton’s jelly (UC-MSCs), placenta, and dental pulp, each exhibiting distinct biological characteristics [12]. These MSC populations vary substantially in their proliferative capacity, multilineage differentiation potential, and secretory profiles, reflecting their tissue-specific adaptations. BM-MSCs, traditionally isolated through invasive bone marrow aspiration, display moderate in vitro expansion kinetics and were originally employed in bone tissue engineering applications, though their harvest yield shows an inverse correlation with donor age [44]. In contrast, AT-MSCs obtained via minimally invasive lipoaspiration procedures demonstrate superior angiogenic potential through robust secretion of trophic factors, including vascular endothelial growth factor (VEGF), transforming growth factor-β1 (TGF-β1), and angiopoietin (ANG), making them particularly suitable for vascularized tissue regeneration [45]. UC-MSCs emerge as an exceptionally promising source due to their unique advantages of low immunogenicity, absence of ethical constraints, minimal tumorigenic potential, and remarkable osteogenic capacity, which is beneficial for bone repair applications [46]. Importantly, MSC populations exhibit source-dependent metabolic specialization, with AT-MSCs primarily utilizing fatty acid oxidation pathways while UC-MSCs preferentially employ glycolytic metabolism. These fundamental metabolic differences may critically determine cellular survival and functional maintenance post-transplantation. Optimal MSC source selection must, therefore, integrate multiple considerations, including the specific therapeutic requirements (such as angiogenesis promotion, immunomodulation, or hard tissue regeneration), donor tissue accessibility, and functional specialization [47,48,49]. Future research directions should focus on elucidating how native tissue microenvironments imprint MSC functional properties to enable precision matching of MSC sources with clinical applications [50,51,52].

### 3.2. Homing Capacity and Drug Delivery

**Figure 2:**
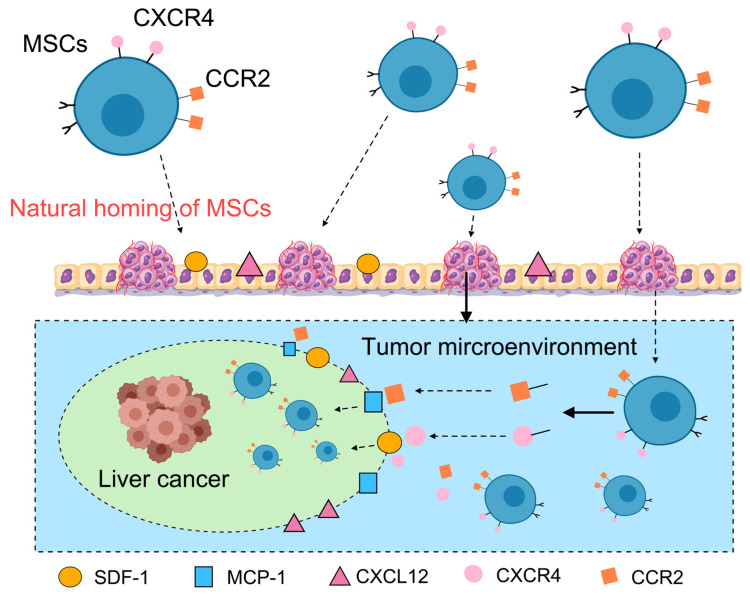
Mechanism of natural homing of mesenchymal stem cells to the tumor microenvironment. This illustration captures the natural homing journey of mesenchymal stem cells (MSCs, in blue) to the hepatocellular carcinoma microenvironment. Originating in the surrounding tissue (top), MSCs express receptors like CXCR4 and CCR2. This directed migration is propelled by chemoattractant gradients (SDF-1, MCP-1, and CXCL12), guiding the cells to the tumor site (bottom, light blue).

Mesenchymal stem cells possess a remarkable capacity for directional migration toward specific tissues or organs, a phenomenon mechanistically similar to lymphocyte homing and, consequently, termed “homing” [53]. This ability relies on synergistic interactions of multiple molecular signaling pathways, enabling MSCs to actively migrate toward tumor tissues and accumulate at tumor sites. The homing mechanism is facilitated by MSC surface expression of key chemokine receptors, including CXCR4 and CCR2, which detect and respond to chemotactic gradients established by tumor-secreted factors such as SDF-1, MCP-1, and CXCL12 within the tumor microenvironment (Figure 2) [54]. Owing to these intrinsic tumor-tropic properties, MSCs are exceptionally promising biological vectors for enhancing precision in anticancer drug delivery systems [55].

Xu et al. provided compelling evidence that MSCs specifically migrate to injured hepatic tissue through the CXCR4/SDF-1 chemotactic axis, with their homing efficiency being substantially amplified in fibrotic liver conditions, thereby opening new avenues for therapeutic enhancement in liver disease management [56]. Yu et al. further expanded this paradigm by demonstrating that MSC homing capability can be significantly augmented through various preconditioning strategies, including hypoxia exposure, pharmacological pretreatment, or targeted genetic modifications [57]. These innovative approaches effectively overcome critical limitations in MSC-based therapies, such as poor in vivo survival rates and suboptimal homing efficiency, thereby substantially improving the therapeutic potential of MSC transplantation for treating hepatic pathologies.

### 3.3. Loading Versatility of MSCs

MSCs and their derived exosomes have emerged as highly adaptable biological delivery systems for cancer treatment, showcasing exceptional functional diversity through multiple therapeutic cargo-loading approaches [55]. MSCs inherently target tumor microenvironments, enabling direct delivery of chemotherapeutics such as doxorubicin. Genetic engineering further allows MSCs to express prodrug-converting enzymes, achieving localized drug activation within tumors [54]. The phospholipid bilayer structure of MSC-derived exosomes provides an ideal vehicle for encapsulating hydrophobic compounds such as paclitaxel, enabling efficient payload delivery through membrane fusion mechanisms [58,59]. For macromolecular therapeutics, MSCs-derived exosomes serve as natural carriers of regulatory nucleic acids, including tumor-suppressing miR-1827, and genetically modified exosomes can function as sustained-release platforms for therapeutic proteins like cytokines [60]. Notably, MSCs act as “Trojan horses” for oncolytic viruses, shielding adenoviruses from immune clearance to enhance tumor targeting [61,62,63]. Innovative dual-delivery strategies combine chemotherapeutics with oncolytic viruses or coordinate exosome-mediated miRNA transfer with conventional cytotoxic drugs to combat therapeutic resistance [64,65,66]. While challenges remain in large-scale production and targeting refinement, emerging technologies, including surface engineering and 3D culture systems, are rapidly advancing MSC and exosome platforms toward intelligent delivery systems that integrate tumor-specific homing, enhanced safety profiles, and multi-agent loading capabilities, positioning them at the forefront of next-generation anticancer strategies. The direct loading method for MSCs primarily uses chemical transfection and electroporation to introduce drugs or gene carriers into mesenchymal stem cells, serving as a key technology for constructing drug delivery systems [55]. Chemical transfection is simple to operate but limited by cell type variability and potential toxicity, while electroporation offers high efficiency but requires precise parameter control to avoid cell membrane damage. Future efforts should focus on developing novel transfection carriers and optimizing protocols to enhance drug loading efficiency while maintaining cell viability and function, thereby advancing clinical translation [54].

### 3.4. Immunomodulatory Properties of MSCs

**Figure 3:**
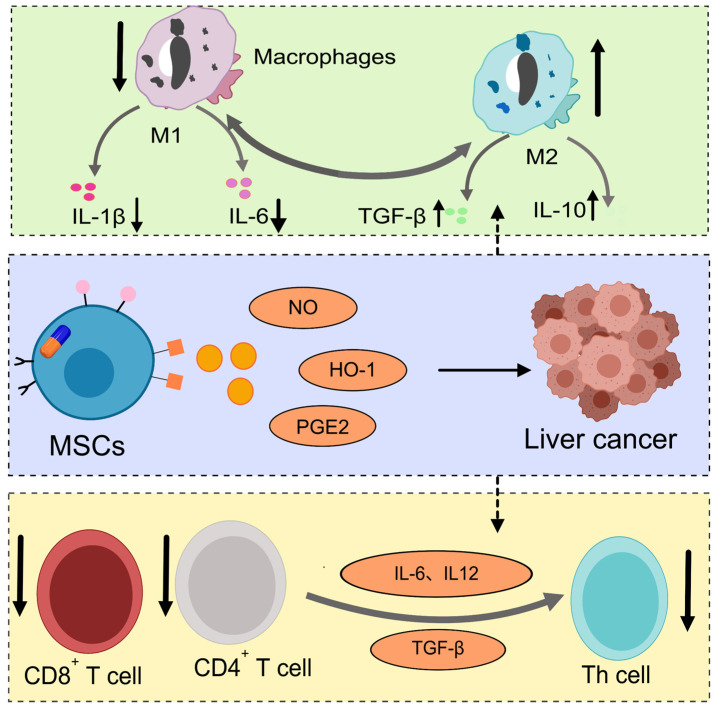
Immunomodulatory effect of MSCs in the hepatocellular carcinoma microenvironment. This schematic diagram unravels the cytokine-mediated interaction network within the hepatocellular carcinoma microenvironment regulated by MSCs. The upper section (light green) details the macrophage dynamic: pro-inflammatory M1 cells release IL-1β and IL-6, while M2 cells produce IL-10, modulated by TGF-β’s bidirectional action. The lower section (light yellow) reveals how immunoregulatory molecules (NO, HO-1, and PGE-2) from MSCs target liver cancer cells, while T cells (CD8^+^, CD4^+^, and Th) contribute cytokines, including IL-6, IL-12, and TGF-β.

MSCs possess remarkable immunomodulatory capabilities and influence the tumor microenvironment (TME) through multiple mechanisms, which demonstrates their dual regulatory characteristics in cancer therapy. By secreting cytokines, exosomes, and other bioactive factors, MSCs contribute to the reprogramming of the TME: they can promote the proliferation and activation of anti-inflammatory immune cells, while under specific conditions, and also initiate pro-inflammatory immune responses, which synergistically inhibit tumor progression (Figure 3) [67]. Regarding their immunosuppressive functions, MSCs produce molecules such as nitric oxide (NO) and heme oxygenase-1 (HO-1) that directly act on T cells, suppressing CD8^+^ T cell cytotoxicity and modulating the differentiation of helper T cells (Th cells) [68]. Furthermore, MSCs interact with macrophages to regulate their polarization state, driving a transition from the pro-inflammatory M1 phenotype to the anti-inflammatory M2 phenotype, thereby enhancing immunoregulatory functions [69]. For instance, MSCs generate immunosuppressive molecules and metabolites, including prostaglandin E2, lactate, and spermidine, to facilitate this shift toward an anti-inflammatory macrophage profile [68]. In terms of immune activation, MSCs promote T cell infiltration into tumor sites, significantly restraining tumor growth—an effect potentially linked to high levels of anti-inflammatory cytokines such as IL-10 in the TME, inducing a pro-inflammatory phenotype in MSCs [70]. Conversely, in low IFN-γ conditions, MSCs exhibit antigen-presenting capacity, activating antigen-specific CD8^+^ T cells and amplifying immune responses [71]. MSCs also secrete IFN-β to directly inhibit tumor cell proliferation [72]. Overall, the immunomodulatory behavior of MSCs is highly context-dependent: during early inflammation, they primarily mediate antigen presentation and immune activation to suppress tumor growth; whereas under sustained inflammatory conditions, they tend to exhibit immunosuppressive properties that may even promote tumor progression [73,74]. This dynamic and plastic immunoregulatory ability highlights the significant potential of MSCs in hepatocellular carcinoma treatment, offering novel strategies and therapeutic promise for precise modulation of the hepatic immune microenvironment to inhibit tumor growth and metastasis.

MSCs exhibit intrinsic immune privilege due to their low immunogenicity and active immunomodulatory capacity [75,76]. Their surface lacks expression of MHC class II molecules and co-stimulatory markers such as CD80 and CD86, enabling evasion of immune recognition and rejection [77,78]. Simultaneously, MSC-secreted immunosuppressive factors, including transforming growth factor-β, prostaglandin E2, and indoleamine 2,3-dioxygenase, regulate diverse immune cell functions [79,80]. This immunomodulation is context-dependent, becoming selectively amplified at pathological sites under inflammatory cytokine stimulation, such as interferon-gamma and tumor necrosis factor-α, thereby minimizing systemic side effects [81]. The tumor-targeting capacity of MSCs positions them as ideal drug delivery vehicles that are capable of transporting oncolytic viruses, chemotherapeutics, or immunomodulators across vascular barriers to lesion sites while also shielding therapeutic payloads from host immune clearance due to their immune-privileged properties [71]. Furthermore, MSC-derived exosomes and apoptotic vesicles inherit the immunomodulatory functions of parental cells while mitigating risks associated with live-cell transplantation [82]. These nanoscale vesicles deliver bioactive cargo such as miRNAs, mRNAs, and proteins to reprogram recipient cell metabolism and immune activity. During tissue repair, MSCs promote regeneration via mitochondrial transfer and secretion of pro-angiogenic factors while reshaping local immune microenvironments—for instance, suppressing B cell-mediated profibrotic activity to alleviate hepatic fibrosis [83]. Three-dimensionally cultured MSC spheroids further amplify these functionalities, establishing MSCs as multifunctional platforms that integrate immune privilege, targeted delivery, and therapeutic potency [84].

### 3.5. Safety and Reliability of MSCs

MSCs demonstrate an exceptional safety profile as therapeutic delivery vehicles, with extensive preclinical and clinical evidence supporting their non-tumorigenic characteristics [85]. The inherent biological properties of MSCs, including limited telomerase activity and finite replicative potential following in vitro expansion, fundamentally prevent malignant transformation [85]. Comprehensive genomic analyses of bone marrow-derived MSCs have confirmed remarkable genetic stability, showing undetectable telomerase activity and negligible oncogenic mutation frequencies even after prolonged culture exceeding 20 passages [44]. The immunological safety of MSCs is ensured by their naturally low immunogenicity, characterized by minimal surface expression of MHC class II molecules and absence of co-stimulatory signals (CD80/CD86), which collectively prevent allogeneic rejection responses [86,87,88]. Advanced genetic engineering approaches now permit precise temporal and spatial control of MSC activity; for example, the Tet-On inducible system, when coupled with the HSV-TK suicide gene, enables targeted elimination of administered MSCs through ganciclovir activation, achieving statistically significant tumor regression (72% volume reduction, *p* < 0.001) while maintaining precise therapeutic control [89]. A comprehensive meta-analysis of clinical data involving 3268 patients receiving MSC therapies revealed an outstanding safety record, with severe adverse events occurring in <0.1% of cases and no reported incidents of tumorigenesis or pathological ectopic tissue formation. This evidence solidifies the clinical viability of MSC-based therapeutic platforms [90]. These intrinsic safety features, combined with emerging engineering strategies, position MSCs as uniquely suited for clinical translation in regenerative medicine and targeted therapy applications. However, caution must be exercised as improper use of MSCs, for instance, through prolonged in vitro expansion or excessive infusion into tumor sites, may promote tumor growth by facilitating angiogenesis or suppressing immune surveillance via paracrine mechanisms [70]. Research indicates that within specific tumor microenvironments, MSCs can secrete factors including VEGF and IL-6, which may indirectly accelerate tumor progression [74]. Consequently, clinical applications require rigorous assessment of cell quality, infusion dosage, and the target tissue microenvironment to minimize these potential risks.

## 4. Advances in MSC-Based Drug Delivery Systems for HCC Therapy

Mesenchymal stem cells (MSCs) and their derived exosomes (MSC-Exos) demonstrate unique potential for drug delivery in the treatment of hepatocellular carcinoma. Studies have shown that MSC-Exos, as natural nanocarriers, can efficiently load chemotherapeutic agents, such as doxorubicin, and evade phagocytosis through their surface proteins, enabling prolonged circulation and targeted delivery [91,92]. These properties position MSC-mediated drug delivery systems as a promising breakthrough strategy for targeted therapy of liver cancer. Compared to macrophage-based carriers, MSCs exhibit a superior capacity to resist the immunosuppressive tumor microenvironment, thereby maintaining more stable homing and penetration efficiency into tumor tissues [54]. Furthermore, MSCs possess longer circulation time and reduced susceptibility to clearance by the mononuclear phagocyte system, enabling more sustained and effective drug delivery to hepatocellular carcinoma sites.

### 4.1. MSC-Mediated Chemotherapy Delivery for Liver Cancer

Mesenchymal stem cells (MSCs) exhibit distinct advantages as delivery vehicles for chemotherapeutic agents in the treatment of hepatocellular carcinoma. A representative strategy involves targeting activated hepatic stellate cells (aHSCs), which highly express platelet-derived growth factor receptor β (PDGFRB) [93]. Engineering MSCs with surface modification of PDGFRB ligands significantly enhances their homing efficiency to fibrotic and tumor microenvironments. These modified MSCs can effectively deliver chemotherapeutic drugs, such as doxorubicin (DOX), to hepatocellular carcinoma tissues, thereby overcoming chemoresistance mediated by drug efflux through P-glycoprotein (P-gp) [94]. Furthermore, placenta-derived MSCs (PD-MSCs) modified with the WKYMVm peptide demonstrate enhanced drug-carrying capacity and exhibit synergistic anti-fibrotic and anti-tumor effects in models combining liver cirrhosis and hepatocellular carcinoma [95]. Recent studies have also revealed that extracellular vesicles secreted by MSCs, such as SIRP-EVs, can co-deliver chemotherapeutic agents and regenerative factors. These vesicles not only block CD47-mediated immune evasion in hepatocellular carcinoma cells but also promote liver repair by reprogramming macrophages [96]. Notably, senescent endothelial cells in the hepatocellular carcinoma microenvironment recruit MSCs via IGF2/MAPK signaling, and drug-loaded MSCs can reverse this pro-tumor microenvironment and suppress the epithelial–mesenchymal transition (EMT) process [97]. Collectively, these findings underscore the potential of MSC-based targeted delivery systems as a novel strategy beyond conventional chemotherapy for hepatocellular carcinoma.

As summarized in Table 1, the loading versatility of MSCs enables a multi-pronged attack on HCC, encompassing direct cytotoxicity, sensitization to chemotherapy, reversal of immunosuppression, and modulation of key oncogenic signaling pathways.

### 4.2. Exosomes and Drug Delivery

Mesenchymal stem cell-derived exosomes (MSC-exosomes) have emerged as highly promising drug delivery vehicles due to their unique biological properties [111]. These 40–160 nm vesicles exhibit inherent biocompatibility and low immunogenicity, with their phospholipid bilayer structure effectively protecting encapsulated therapeutics [112]. For instance, desialylated MSC exosomes achieve efficient doxorubicin delivery by targeting the asialoglycoprotein receptor on hepatocellular carcinoma cell surfaces, significantly enhancing tumor-specific drug accumulation while mitigating cardiotoxicity [91]. Exosomes from diverse MSC sources, including bone marrow, adipose tissue, and umbilical cord, display distinct proteomic profiles but share characteristic surface markers such as CD9 and CD63 [50]. They deliver functional miRNAs that modulate tumor signaling pathways: adipose-derived MSC exosomes carrying miR-199a-3p enhance sorafenib sensitivity in HCC cells by suppressing mTOR signaling, while other miRNAs like miR-100 and miR-143 inhibit tumor progression through key regulatory pathways [113]. Compared to live-cell therapies, MSC exosomes retain the ability to cross biological barriers without posing tumorigenic risks, thus offering an ideal platform for developing safe and precisely targeted therapies [99,114].

Pascucci et al. first demonstrated that paclitaxel-primed MSCs acquire potent antitumor activity by packaging and delivering active drugs via extracellular vesicles, positioning MSCs as bioreactors for targeted anticancer agents [115,116]. Rajashekhar et al. further validated that noscapine-loaded MSC exosomes exhibit enhanced cellular uptake efficiency and broad-spectrum antitumor effects across cancer types [100,117]. Meanwhile, preclinical studies have underscored their notable therapeutic potential: for instance, Bruno et al. reported that exosomes derived from adipose tissue MSCs not only suppress HCC growth in rat models and improve apparent diffusion coefficient values, but also amplify natural killer T cell-mediated antitumor responses and demonstrate histopathological efficacy [103,118].

MSC-exosomes serve as highly efficient nucleic acid delivery vehicles, capable of carrying diverse functional nucleic acid molecules (e.g., miRNAs and lncRNAs) to precisely regulate key signaling pathways involved in tumorigenesis and progression, demonstrating substantial potential for cancer treatment. For instance, exosome-delivered miR-199a-3p and miR-122 can specifically inhibit the mTOR and PKM2 signaling pathways, respectively, and downregulate the expression of multidrug resistance-associated proteins such as P-gp and BCRP, thereby reversing chemoresistance in tumor cells [99,119,120]. In the context of hepatocellular carcinoma treatment, exosomes derived from miR-122-transfected MSCs efficiently deliver this miRNA into cancer cells, markedly enhancing their sensitivity to sorafenib [106]. Furthermore, exosome-mediated transfer of lncRNAs such as MALAT1 modulates DNA damage repair mechanisms and increases tumor cell radiosensitivity [121]. Optimization strategies, such as strontium pretreatment, can markedly enhance the loading efficiency of miRNAs in synovial MSC-derived exosomes, enabling the enrichment of specific therapeutic miRNAs and amplifying their antitumor effects [122]. Meanwhile, engineering approaches developed exosomes overexpressing miR-486-5p, which effectively inhibit radiation-induced ferroptosis and improve normal tissue radiotolerance; similarly, while exosome-enriched miR-466f-3p suppresses the AKT/GSK3β signaling pathway to reverse the epithelial–mesenchymal transition process, thereby enhancing tumor response to radiotherapy [123]. These findings highlight MSC-exosomes as a natural, efficient, and engineerable nucleic acid delivery system with broad prospects for application in cancer genetic therapy.

### 4.3. MSC-Delivered Immunomodulators for Cancer Therapy

In the context of immunotherapy, MSC-exosomes serve as a promising delivery platform for remodeling the tumor immune microenvironment (TIME). They are capable of transporting key bioactive agents such as TGF-β antagonists and immune checkpoint modulators (e.g., PD-L1 degraders). For instance, loading PD-L1 siRNA or small-molecule degraders has been shown to reverse the immunosuppressive state in hepatocellular carcinoma models post-radiofrequency ablation (iRFA), while simultaneously restoring the anti-tumor function of cytotoxic T cells [124]. Meanwhile, delivering TGF-β receptor inhibitors via MSC-exosomes can block the immunosuppressive signaling cascades triggered by the TGF-β pathway. This not only significantly enhances the infiltration of cytotoxic T cells into the tumor site but also boosts their activation, thereby effectively overcoming resistance to immunotherapy [125].

Recent research has shed light on additional mechanisms underlying immunotherapy resistance, providing new directions for MSC-exosome-based interventions [98]. A study by Lu et al. demonstrated that the interaction between tumor-associated macrophages and tumor cells drives the amplification of the spatially isolated adenosine (ATP-ADO) pathway, leading to the production of large amounts of immunosuppressive adenosine, which induces T cell exhaustion and ultimately contributes to resistance to anti-PD1 therapy in HCC [126]. This finding underscores that targeting the adenosine pathway is a pivotal strategy for reversing immunotherapy resistance. Building on this insight, MSCs-exosomes can be engineered into “biological missiles” to disrupt the ATP-ADO pathway [14]. By loading these carriers with CD39/CD73 inhibitors (which block adenosine generation at its source) or A2AR antagonists (which inhibit adenosine-mediated immunosuppressive signaling), MSC-exosomes can effectively alleviate adenosine-induced suppression of cytotoxic T lymphocytes (CTLs) and restore their tumor-killing capacity, exerting a synergistic effect with anti-PD-1/PD-L1 therapy, further enhancing the overall anti-tumor immune response [127]. The MSC-based delivery strategy combines excellent tumor-homing ability, biocompatibility, and low systemic toxicity, offering a transformative and promising approach for overcoming immunotherapy resistance mediated by tumor-immune microenvironment interactions.

### 4.4. Breakthroughs in Genetically Engineered MSC Technologies

Concurrently, genetic engineering of MSCs has achieved transformative progress. When engineered as “Trojan horses” for oncolytic adenoviruses (e.g., Ad5/3 chimera), MSCs exploit their intrinsic tumor tropism to overcome viral clearance barriers. In HCC models, this approach not only boosts viral delivery efficiency by multiple times but also mitigates hepatic enzyme abnormalities [62,110]. For radiation-responsive MSCs engineered with the SMAD-NIS system, their ability to enhance radiotherapy efficacy operates through two synergistic mechanisms: On one hand, TGF-β1-activated SMAD signaling strengthens the tumor-targeted migration of MSCs; on the other hand, the expression of the sodium-iodide symporter (NIS) drastically elevates the uptake of iodine-131 (^131^I) in tumors. When combined with radiotherapy, this strategy achieves a nearly 50% complete remission rate [128]. These innovative strategies integrate the precision of exosome-mediated delivery with the programmable features of engineered MSCs, establishing next-generation intelligent carrier platforms for hepatocellular carcinoma therapy [62,128].

CXCR4 serves as a critical chemokine receptor on the surface of mesenchymal stem cells, while its ligand stromal cell-derived factor 1 (SDF-1) is highly expressed in injured or inflamed regions, including tumors, ischemic tissues, and bone marrow microenvironments [129]. This spatial distribution creates a concentration gradient that drives the directional migration of CXCR4-expressing MSCs [130,131]. For example, genetically engineered MSCs overexpressing CXCR4 demonstrate enhanced antioxidant stress capacity and improved tumor-targeted migration, which is regulated by the transcription factor nuclear factor erythroid 2-related factor 2 (Nrf2) [132]. Moreover, during the progression of HCC, increased matrix stiffness activates CXCR4 signaling, which in turn further accelerates tumor progression. By serving as a mediator of mechanical stimuli, CXCR4 may strengthen the interactions between MSCs and the tumor matrix, thereby promoting the enrichment of MSCs within HCC tissues [133]. Notably, chemotactic processes are governed by multi-receptor networks, rendering single-gene editing insufficient. To address this limitation, co-editing of CCR2 (a receptor responsive to MCP-1 signaling) and CXCR7 (a co-receptor for SDF-1) can broaden the responsiveness of human MSCs to a wider spectrum of chemokines. This co-editing strategy also effectively mitigates off-target migration, a problem that may otherwise arise from the excessive activation of individual signaling pathways [134].

**Figure 4:**
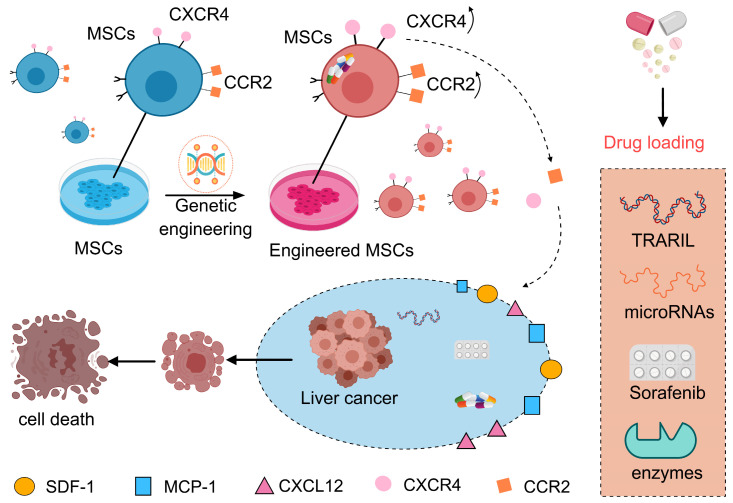
Mechanism of engineered MSC-mediated targeted drug delivery for hepatocellular carcinoma therapy. This schematic diagram illustrates the mechanism of targeted drug delivery using mesenchymal stem cells (MSCs) for hepatocellular carcinoma (HCC) therapy. The process begins when HCC cells secrete chemoattractant factors (e.g., SDF-1), which bind to corresponding receptors (e.g., CXCR4) on MSCs, initiating a homing response. Subsequently, these MSCs, which can be genetically engineered and loaded with therapeutic agents (TRAIL, miRNAs, etc.), migrate along the concentration gradient to specifically target the tumor site. Finally, upon arrival, the MSCs release their therapeutic payload directly into the tumor microenvironment, showcasing a sophisticated cell-based delivery strategy.

Engineered MSCs demonstrate unique advantages as drug delivery carriers, with their core value rooted in significantly enhanced targeting specificity and therapeutic efficacy through genetic modification and bioengineering technologies [135]. In drug loading, modified MSCs such as TRAIL, interleukin-10, microRNAs, or prodrug-converting enzymes can achieve precise localized release via CXCR4-mediated tumor-homing mechanisms [55,105,109,136]. For instance, MSCs engineered to express both CXCR4 and interleukin-10 exert robust anti-inflammatory effects in graft-versus-host disease models. Meanwhile, MSCs overexpressing CXCR4, when loaded with chemotherapeutic agents such as paclitaxel, not only preserve their inherent tumor tropism but also substantially boost drug accumulation within the tumor microenvironment (Figure 4) [132,137].

The integration of light- or heat-responsive genetic switches provides innovative solutions for spatiotemporally precise regulation of drug release kinetics [138]. In optogenetic systems, the LightOn system enables high dynamic-range gene expression upon blue light activation, which has been successfully applied for controlled spatiotemporal delivery of diphtheria toxin A fragment-encoding plasmids within tumor microenvironments [139]. Beyond optogenetics, visible light-responsive polymers leverage their photodegradation properties to dynamically remodel microenvironments and modulate VEGF gene activation modules [140]. Additionally, novel light-switchable DNA conformation technologies address the limitation of slow reversible switching kinetics through intramolecular triplex designs, substantially enhancing response speeds in photoregulated chemical processes [141]. For thermoresponsive mechanisms, magnetic micro-organogels with dual sensitivity to temperature and glutathione achieve synergistic controlled release. Moreover, hydrogels based on N-isopropylacrylamide (NIPAM) exploit their lower critical solution temperature (LCST) characteristics to trigger precise drug release at body temperature [142,143,144]. As an example, pH-sensitive photothermal-responsive nanocarriers, such as doxorubicin-loaded HPCF, are able to release doxorubicin under acidic tumor microenvironments and near-infrared irradiation, amplifying efficacy via light-controlled genetic switches [145].

## 5. Conclusions and Future Perspectives

MSCs stand out as superior drug delivery vehicles, largely attributed to their distinctive biological characteristics. A key advantage lies in their intrinsic homing ability via chemokine signaling axes such as CXCR4/SDF-1, which enables active and precise targeting of hepatocellular carcinoma (HCC) tissues [146]. Notably, animal studies have confirmed that the homing efficiency of MSCs is three times higher than that of unmodified cells, underscoring their targeting superiority. Among the major sources of MSCs, including bone marrow, adipose tissue, and umbilical cord, umbilical cord-derived MSCs (UC-MSCs) exhibit the greatest potential for clinical translation. This is primarily due to their two critical merits: extremely low immunogenicity (which minimizes the risk of immune rejection) and complete avoidance of ethical controversies (a common limitation associated with other stem cell sources) [75]. Drug-loading techniques such as electroporation, chemical transfection, and membrane fusion allow efficient encapsulation of chemotherapeutic agents like doxorubicin and paclitaxel, nucleic acid therapeutics such as miR-122 and siRNA, and immunomodulators including interleukin-12 [71,101]. MSC-derived exosomes, with their 40–160 nm nanostructure and inherent biocompatibility, effectively penetrate the blood–tumor barrier, achieving significantly higher drug accumulation in tumor cores compared to conventional formulations [147].

Genetic engineering tools such as CRISPR/Cas9 enable precise modulation of homing-related genes like CXCR4, improving tumor-targeting efficiency by two to threefold. Similarly, light- and heat-responsive systems achieve spatiotemporal drug release [148]. Surface modifications of exosomes with targeting ligands such as ASGPR enhance specificity, with preclinical studies showing markedly increased tumor uptake.

Despite these advances, critical challenges persist in the development of MSC-based therapies. MSCs exhibit dual-edged effects during tumor progression—their derived exosomes may promote angiogenesis via factors like EPHA2 or foster pre-metastatic niches through miR-194/215 clusters [14,84,149,150]. The hypoxia microenvironment in tumor tissue upregulates H19/MMP1 in MSC exosomes to accelerate metastasis, while external stimuli such as nicotine alter immune microenvironments [151]. Scaling exosome production faces hurdles in yield and heterogeneity, though advances in preconditioning, nanomaterial-assisted methods, and 3D culture show promise [152]. Additionally, regulatory gaps in GMP compliance and standardized exosome quantification further complicate the clinical translation of MSC-based drug delivery systems [11].

Future research should prioritize several key avenues to advance mesenchymal stem cell (MSC)-based therapies for hepatocellular carcinoma (HCC). First, the application of single-cell sequencing technologies is crucial to precisely identify and isolate functionally distinct MSC subpopulations, thereby enabling the purification of cells with optimal therapeutic potential. Second, AI-driven optimization of drug-loading protocols can enhance the efficiency and precision of encapsulating therapeutic agents into MSCs while preserving their viability and homing capabilities [153,154]. Third, the development of sophisticated organoid-MSC co-culture models will provide a more physiologically relevant platform for pre-clinical testing, allowing for accurate prediction of treatment efficacy and toxicity in a human-mimetic microenvironment. Furthermore, the integration of MSC-based delivery systems with other cutting-edge modalities, such as CAR-T cell therapy and epigenetic modulators, could lead to synergistic anti-tumor effects and unlock novel combination treatment paradigms for HCC [102,155]. For instance, MSCs could be engineered to deliver immunomodulatory signals that enhance CAR-T cell persistence or to target epigenetic alterations in the tumor microenvironment. Finally, with sustained innovation in gene editing tools (e.g., CRISPR-Cas9) and advanced drug delivery technologies, MSC-based platforms are poised to become highly versatile vehicles for targeted therapy. These advancements hold considerable promise for advancing precision and personalized medicine, ultimately improving the clinical management and outcomes for patients with hepatocellular carcinoma [104,107,108,156].

## Figures and Tables

**Table 1:** Therapeutic agents loaded into mesenchymal stem cells for hepatocellular carcinoma therapy.

Source	Therapeutic Strategy	Synergistic Drug/Method	Results	Reference
Adipose tissue-derived MSCs (AT-MSCs)	Exosome-mediated miR-122 delivery	Sorafenib	Exosomes regulated target gene expression via miR-122, enhancing hepatocellular carcinoma (HCC) sensitivity to chemotherapy.	[98]
	Exosome-mediated miR-199a delivery	Doxorubicin	Significantly improved HCC chemosensitivity by inhibiting the mTOR pathway and reduced tumor volume in vivo.	[99]
	Cell-based therapy	Radiotherapy	Inhibited HCC cell growth, migration, and invasion.	[16]
Bone marrow-derived MSCs (BMSCs)	Measles virus infection (oncolytic viral vector)	None	Measles virus-infected MSCs evaded immune clearance, reduced tumor volume, and prolonged survival in HCC models without systemic immune reactions.	[63]
	Exosome-encapsulated norcantharidin (NCTD)	None	Exosomes acted as drug carriers, enhancing antitumor effects against HCC.	[100]
	Exosome-delivered GRP78-targeting siRNA	Sorafenib	Reversed sorafenib resistance in HCC and significantly reduced tumor volume while prolonging survival in murine models.	[101]
	T cell-activating MSCs	CAR-T cell therapy	Enhanced specific cytotoxicity against HCC via Glypican-3-targeted CAR-T cells.	[102]
	Cell-based therapy	None	Exosomes modulated cancer stemness in HCC, suppressing tumor growth.	[103]
Human umbilical cord MSCs (hUC-MSCs)	Conditionally replicative adenovirus (CRAd) delivery	None	Liver differentiation-dependent viral release enabled specific HCC cell elimination with minimal hepatotoxicity.	[104]
	AFP promoter-driven sTRAIL expression	5-FU	Suppressed orthotopic HCC tumor growth, extended median survival, and caused no significant hepatic/renal toxicity.	[105]
	Exosome-mediated miR-499a-5p delivery	None	Attenuated liver fibrosis by targeting ETS1/GPX4-mediated ferroptosis in hepatic stellate cells, potentially preventing HCC progression.	[66]
MSCs (unspecified source)	Cell-based therapy	Sorafenib	Combined therapy suppressed HCC proliferation, reduced angiogenesis, and induced apoptosis while maintaining MSC-mediated anti-inflammatory effects.	[106]
	Oncolytic adenovirus delivery	Oncolytic virotherapy	Enhanced antitumor efficacy against HCC while preventing hepatotoxicity.	[62]
	IL-12 genetic modification	Prophylactic monotherapy	Demonstrated significant cancer prevention efficacy in three unestablished tumor models, including HCC.	[107]
	Tissue-specific suicide gene system	Ganciclovir	Targeted HCC stromal microenvironment selectively, suppressing tumor growth.	[74]
	HNF4α overexpression	None	Suppressed HCC development via downregulation of Wnt/β-catenin signaling.	[108]
	TRAIL genetic modification	Cisplatin	MSCs as TRAIL delivery vehicles enhanced apoptosis induction in HCC cells and synergized with cisplatin to inhibit tumor growth.	[109]
	Apoptin genetic modification	None	Induced HCC apoptosis significantly, reduced tumor weight in animal models without systemic toxicity.	[110]

## Data Availability

No new data were created or analyzed in this study.
